# RNA N6-methyladenosine demethylase FTO promotes breast tumor progression through inhibiting BNIP3

**DOI:** 10.1186/s12943-019-1004-4

**Published:** 2019-03-28

**Authors:** Yi Niu, Ziyou Lin, Arabella Wan, Honglei Chen, Heng Liang, Lei Sun, Yuan Wang, Xi Li, Xiao-feng Xiong, Bo Wei, Xiaobin Wu, Guohui Wan

**Affiliations:** 1 School of Pharmaceutical Sciences, Sun Yat-Sen University, Guangzhou, 510006 China; 2 The Eighth Affiliated Hospital, Sun Yat-Sen University, Shenzhen, 518033 China; 3 The Third Affiliated Hospital, Sun Yat-Sen University, Guangzhou, 510630 China; 4 The First Affiliated Hospital, Sun Yat-Sen University, Guangzhou, 510630 China; 5 The Sixth Affiliated Hospital, Sun Yat-Sen University, Guangzhou, 510655 China; 6 School of Pharmaceutical Sciences, the Fifth Affiliated Hospital, Guangzhou Medical University, Guangzhou, 511436 China; 7 Guangdong Key Laboratory of Chiral Molecule and Drug Discovery, School of Pharmaceutical Sciences, Sun Yat-Sen University, Guangzhou, 510006 China

**Keywords:** Breast cancer, N6-methyladenosine, FTO, BNIP3, Apoptosis

## Abstract

**Background:**

N6-methyladenosine (m6A) modification is the most pervasive modification in mRNA, and has been considered as a new layer of epigenetic regulation on mRNA processing, stability and translation. Despite its functional significance in various physiological processes, the role of the m6A modification involved in breast cancer is yet fully understood.

**Methods:**

We used the m6A-RNA immunoprecipitation sequencing to identify the potential targets in breast cancer. To determine the underlying mechanism for the axis of FTO-BNIP3, we performed a series of in vitro and in vivo assays in 3 breast cancer cell lines and 36 primary breast tumor tissues and 12 adjunct tissues.

**Results:**

We showed that FTO, a key m6A demethylase, was up-regulated in human breast cancer. High level of FTO was significantly associated with lower survival rates in patients with breast cancer. FTO promoted breast cancer cell proliferation, colony formation and metastasis in vitro and in vivo. We identified BNIP3, a pro-apoptosis gene, as a downstream target of FTO-mediated m6A modification. Epigenetically, FTO mediated m6A demethylation in the 3’UTR of BNIP3 mRNA and induced its degradation via an YTHDF2 independent mechanism. BNIP3 acts as a tumor suppressor and is negatively correlated with FTO expression in clinical breast cancer patients. BNIP3 dramatically alleviated FTO-dependent tumor growth retardation and metastasis.

**Conclusions:**

Our findings demonstrate the functional significance of the m6A modification in breast cancer, and suggest that FTO may serve as a novel potential therapeutic target for breast cancer.

**Electronic supplementary material:**

The online version of this article (10.1186/s12943-019-1004-4) contains supplementary material, which is available to authorized users.

## Background

Breast cancer continues to be a severe threat towards women in the world. It is estimated that more than 2.1 million new cases of breast cancer occurred in 2018, causing 627,000 death in women [1]. With the advent of molecular classification in breast cancer, the 5-year survival rate for patients diagnosed with localized tumors reached 90%, however, for those patient diagnosed with advanced tumors, the 5-year survival rate was less than 30% [2]. The mortality from breast cancer was primarily due to chemo-resistance and metastasis. Administration of novel target intervention may help to prevent initial or aggressive metastasis, thereby improving survival and clinical outcomes for patients with advanced breast tumors. While global gene expression patterns of breast cancer have been extensively studied, post-transcriptional regulation of gene expression involved in advanced breast tumors has yet fully investigated. Examining post-transcriptional gene regulation can better understand the molecular mechanism of breast cancer, providing a more comprehensive picture of the biology of cancer progression.

N6-methyladenosine (m6A) modification is the methylation of the adenosine base at the nitrogen-6 position of mRNA. The m6A modification is the most pervasive internal modification of mRNA in mammalian cells, with wide distribute (> 25%) abundance in the transcripts [3, 4]. Unlike other modifications of mRNA, the m6A modification is dynamically reversible as the DNA and histone modifications [5], and plays an critical role in regulating precursor mRNA maturation, translation and degradation [3, 6, 7]. The m6A modification is mainly mediated by the methyltransferase (METTL3, METTL14 and WTAP) [8–10], demethylase (FTO and ALKBH5) [5, 11] and proteins that preferentially recognized m6A methylated transcripts (YTH domain family proteins [12–16], HNRNPA2B1 [17] and IGF2BP [18]). The m6A modification was found to regulate stem cell differentiation and self-renewal through affecting mRNA turnover during cell differentiation and embryonic development [19, 20]. Emergent evidence has shown that the m6A modification plays an important role in the occurrence and development of various human diseases [21–24].

FTO was the first m6A demethylase to show m6A demethylation activity on mRNA and catalyze m6A demethylation in a ferrous iron dependent manner [5]. Previously, FTO was known to be highly associated with increased body mass and obesity in childhood and adult [25, 26]. FTO-knockout mice showed protection from obesity but caused growth failure [27]. Recently, FTO was shown to mediate mRNA processing of adipogenetic regulatory factors in adipogenesis by m6A demethylation [28]. Chen and He groups showed an oncogenic role of FTO in acute myeloid leukemia (AML) [29]. They found that FTO was high expressed in AML and inhibited ASB2 and RARA by reducing their m6A methylated level in the transcripts, resulting in enhancement of cell transformation and leukemogenesis [29]. The elevated level of FTO was also found in cervical squamous cell carcinoma (CSCC) and glioblastoma [30, 31]. Inactivation of FTO in leukemia induced sensitivity of resistance cells to tyrosine kinase inhibitor [32], indicating that the FTO-m6A axis may serve as novel potential therapeutic target in human cancers [33]. However, the underlying epigenetic regulation of FTO, as m6A demethylase, in breast cancer initiation and progression has yet to be investigated.

In the present study, we sought to determine the role of m6A modification in breast cancer, and investigated the underlying molecular mechanism by which the m6A modification affects the initiation and progression of breast cancer. We first detected the levels of the m6A modification in breast cancer, and observed that FTO was up-regulated in human breast cancer tissues. Next, we demonstrated that silence of FTO could significantly reduce breast cancer cell proliferation, colony formation and enhance cell apoptosis. Further, we identified that BNIP3, a pro-apoptotic member of the Bcl-2 family of apoptotic proteins [34], was the target gene of FTO in mediating breast cancer proliferation and progression. We found that silencing BNIP3 could significantly alleviate FTO-dependent tumor growth retardation in vitro and in vivo. Based on the data, we revealed the important role of m6A modification mediated by FTO in breast cancer, and proposed that FTO may act as a novel therapeutic target in breast cancer progression.

## Methods

### Breast cancer patient samples, cell lines and cell culture

The breast tumor and normal tissues were obtained at the Third Affiliated Hospital of Sun Yat-Sen University and were approved by the institutional review board of the hospital. The study is compliant with all relevant ethical regulations regarding research involving human participants. For fresh tissues, breast tumors and adjacent normal tissues were separately dissected at the time of surgery and immediately transferred to RNAlater (R0901, Sigma). The paraffin-embedded specimens were collected from at the Eighth Affiliated Hospital of Sun Yat-Sen University. Breast cancer cell lines MDA-MB-231, MCF-7 and 4 T1 were obtained from American Type Culture Collection (ATCC) with authentication. These cell lines were cultured in Dulbecco’s modified Eagle’s medium (DMEM, Corning, USA) with 10% fetal bovine serum (Gibco, USA) and antibiotics (Gibco, USA). Cells were grown in a 5% CO2 cell culture incubator at 37 °C.

### Plasmid constructions, cell transfection, and infection

Stable knockdown of target genes was achieved by lentiviral-based short-hairpin RNA (shRNA) delivery. PLKO.1 vector with anti-puromycin or anti-hygromycin plasmid was constructed by using the primer sequences listed in

Additional file 1: Table S1. For YTHDF2 overexpressing system, YTHDF2 cDNA (NM_016258) was cloned into pCDH puro lentiviral vector (CD510B-1, System Biosciences), For shRNA knockdown and overexpression, pLKO.1 and pCDH constructs together with packing and helper plasmids PAX2 and MD2G were co-transfected into 293 T cells by Calcium Phosphate Transfection Kit (CAPHOS-1KT, Sigma). Viruses were collected, filtered, and titrated before infecting target cells with 8 mg/ml Polybrene (TR-1003, Sigma). The infected cells were screened by puromycin or hygromycin accordingly.

### Immunohistochemistry

For immunohistochemistry (IHC) analysis, breast cancer specimens tissue slides were deparaffinized, rehydrated through an alcohol series followed by antigen retrieval with sodium citrate buffer. Tumor sections were blocked with 5% normal goat serum (Vector) with 0.1% Triton X-100 and 3% H_2_O_2_ in PBS for 60 min at room temperature and then incubated with appropriate primary antibodies 4 °C overnight. IHC staining was performed with horseradish peroxidase (HRP) conjugates using DAB detection. Nuclei were counterstained with Hoechst. Images were taken with Nikon microscopy.

### Western blot analysis and antibodies

Cells in 6-well plate or 60 mm^2^ dishes were lysed in 80–100 ul modified RIPA buffer (P0013B, Beyotime, China) containing the complete cocktail of protease inhibitors (#11836153001, Roche, Switzerland). Protein concentrations were determined with the BCA protein assay kit (P0011, Beyotime, China). Proteins were separated by 12 or 15% SDS-PAGE and transferred to nitrocellulose filters, and blotted with related antibodies in 4 °C overnight. Secondary antibodies pre-labeled in room temperature for 1 h. The nitrocellulose filters with target protein were exposed in visualizer (4600, Tanon, China). Antibodies were purchased from the following: anti-FTO (ab124892, 1:1000, Abcam), anti-BNIP3 (ab109362, 1:1000, Abcam), anti-Bcl2 (A0208, 1:1000, ABclonal), anti-β-actin (AF1700, 1:1000, R&D system), anti-tubulin (13E5, 1:1000, Cell Signaling) and anti-caspase 3 (A2156, 1:1000, ABclonal).

### RNA isolation and RT-PCR

Total RNAs were extracted by Trizol (ThermoFisher, USA) following the manufacturer’s instruction. Complementary DNA was synthesized using the SuperScript™ III First-Strand Synthesis System DNA using the PrimeScript RT reagent Kit (RR036A, Takara, Japan). Real-time reverse-transcription PCR was carried out by SYBR-Green Master mix (RR820B, Takara, Japan) in 7500 apparatus (Applied Biosystems). GAPDH was used as an internal control for the normalization. All primers used in this study are listed in Additional file 1: Table S1.

### RNA m6A quantification

Total RNAs were isolated by TRIzol (ThermoFisher, USA) according to the manufacturer’s instructions. RNA quality was analyzed by NanoDrop3000. The EpiQuik m6A RNA Methylation Quantification Kit (Colorimetric) (P-9005, Epigentek, USA) was used to measure the m6A content in total RNAs. Briefly, 200 ng RNAs were coated on assay wells. Capture antibody solution and detection antibody solution were then added to assay wells separately in a suitable diluted concentration following the manufacturer’s instructions. The m6A levels were quantified colorimetrically by reading the absorbance of each well at a wavelength of 450 nm, and calculations were performed based on the standard curve.

### RNA m6A dot blot assay

The poly (A) RNAs (300 ng) were spotted onto a nylon membrane (GE Healthcare). The membranes were then UV cross-linked (254 nm), blocked, incubated with m6A antibody (ABE572, 1:1000, Merck Millipore) in 4° Covernight. Antibodies pre-labeled in room temperature for 1 h. The nylon membrane with m6A dots were exposed in visualizer (4600, Tanon, China). The same 300 ng poly (A) RNAs were spotted on the membrane, stained with 0.2% methylene blue in 0.3 M sodium acetate (pH 5.2) for 2 h, and washed with ribonuclease-free water for 1 h.

### RNA m6A sequence and m6A-RNA immunoprecipitation assay

Total RNAs were extracted by TRizol (ThermoFisher, USA) from stable shFTO MDA-MB-231 cells and the controls. Chemically fragmented RNA (100 nucleotides) was incubated with m6A antibody for immunoprecipitation according to the standard protocol of Magna methylated RNA immune-precipitation (MeRIP) m6A Kit (#17–10,499, Merck Millipore, USA). Enrichment of m6A containing mRNA was analyzed either by qRT-PCR with the primers listed in Additional file 1: Table S1 or by high-throughput RNA sequencing. For high-throughput sequencing, purified RNA fragments from m6A-MeRIP were used for library construction with the NEBNext Ultra RNA library Prep kit for Illumina (E7530S, NEB, USA) and were sequenced by Illumina HiSeq 2000. Library preparation and high-throughput sequencing were performed by Novogene (Guangzhou, China). Sequencing reads were aligned to the human genome GRCh37/hg19 by Bowtie2, and the m6A peaks were detected by magnetic cell sorting as described [35].

### Cell proliferation and cell apoptosis assays

For CCK8 assay, cells were seeded at 1000 cells per well in 96-well plates with fresh medium. Cell viability was assayed using Cell Counting Kit-8 (CK04, Dojindo, Japan) at the time in 0, 48, 72, 96,120,144 h. The microplates were incubated at 37 °C for additional 4 h. Absorbance was read at 450 nm using a microplate reader (ThermoFisher, USA) and the results were expressed as a ratio of the treated over untreated cells (as 100%).

For EdU (5-Ethynyl-2′-deoxyuridine) assay, logarithmic growth stage cells were seeded in 6-well plate with corresponding concentration of EDU reagent for 3 h. Cells were washed with PBS for 5 min twice, before incubating with 4% Paraformaldehyde for 30 min. After washing with PBS for 5 min twice, samples were permeated with 0.3% TritonX-100 in PBS, and dyed with reaction solution (C0075S, Beyotime, China).The images collected with 20× and 40× visions in Nikon microscopy.

For cell apoptosis assays, cells were performed using Annexin V-PI Apoptosis Detection Kit I (WLA001a, Wanleibio, China) according to the manufacturer’s instruction, and followed by flow cytometry analysis (Beckman, USA).

### Mammosphere formation assay and clonogenic assays

For mammosphere formation assay, cells were seeded into ultralow attachment plates (Corning) at a density of 20,000 viable cells/mL in a serum-free DMEM-F12 supplemented with 100× insulin, 20 ng/mL epidermal growth factor and 20 ng/mL basic fibroblast growth factor (Sigma), and 0.4% bovine serum albumin (Sigma), 100 × penicillin &streptomycin for two weeks until the mammosphere became visible.

For clonogenic assay, cells were seeded onto 35 mm^2^ dishes at a density of 5000 viable cells/mL in a serum-free DMEM-F12 supplemented with 100× insulin, 20 ng/mL epidermal growth factor and 20 ng/mL basic fibroblast growth factor (Sigma), and 0.4% bovine serum albumin (Sigma), 100 × penicillin &streptomycin for two weeks until the colonies became visible and stained with Crystal Violet Staining Solution.

### Luciferase reporter assays and mutagenesis assay

The dual-luciferase vector pmiGLO was purchased from Promega (C838A, Promega, USA). The 3’UTR of BNIP3 was amplified by PCR using the genomic DNA from MDA-MB-231 cells as a template. A clone whose sequence was identical to the NCBI reference sequence NM_004052 was used to clone into pmiGLO vector with SacI and SalI restriction sites. Three putative m6A recognition sites were identified in 3’UTR. Mutagenesis from A to T was generated by QuikChange II Site-Directed Mutagenesis Kit (200,523, Agilent, USA) according to the instruction. Luciferase activity was measured by Dual Luciferase Reporter Gene Assay Kit (RG028, Beyotime, China) in GM2000 (Promega). Experiments were performed in triplicates. The firefly luciferase activity values were normalized to the *Renilla* luciferase activity values that reflect expression efficiency. Data are presented as mean values (± s.d.).

### Animal experiments

Mice were housed at five mice per cage under pathogen-free conditions. All animal care and experiments were approved by the Institutional Animal Care and Use Committee of Sun Yat-Sen University, Guangzhou, China, and the study is compliant with all relevant ethical regulations regarding animal research. Mice were euthanized when they met the institutional euthanasia criteria for tumor size and overall health condition.

For the subcutaneous implantation model, 5 4-week-old female Balb/c mice were randomly grouped and injected with 1 × 10^6^ shCtrl, shFTO or shFTO/shBNIP3 KD 4 T1 cells. Tumors were measured with a caliper every 4 days to analyze tumor growth. Tumor volume was calculated by the formula V = ab2/2, where a and b are the tumor’s length and width, respectively. At the experimental endpoint, tumors tissues were harvested and fixed with 4% PFA for paraffin-embedded section.

For tumor metastasis mouse model, 5 4-week-old female Balb/c mice were randomly grouped and injected with 1 × 10^6^ shCtrl, shFTO or shFTO/shBNIP3 KD 4 T1 cells via tail vein. To detect lung metastasis, mice were sacrificed 3 weeks after tumor cells injection. Lung tissues were harvested and fixed with 4% PFA for paraffin-embedded section and lung metastases were detected with the Nikon microscopy.

For orthotopic xenograft mouse model, 5 4-week-old female NOD/SCID mice were randomly grouped. After NOD/SCID were anaesthetized and the skin was incised, shCtrl or shFTO MDA-MB-231-luciferase cells (1 × 10^6^) in 50 ul Hanks solution were orthotopically injected into mammary fat pads using a 1-ml Hamilton microliter syringe, and then the incision was closed using surgery suture threads with needle. Mice tumors were monitored by the IVIS system after luciferin injection for 15 min.

### Bioinformatics analysis

The gene expression profile dataset GSE9014, GSE11812 and GSE3188 was downloaded from GEO database. Data from GEO or RNA-Seq were analyzed by R (V3.3, http://www.bioconductor.org) with edgeR package. Fold-change (FC) of gene expression was calculated with a threshold criteria of log2FC ≥ 1.5 and *P* value< 0.01. KEGG pathway enrichment analysis was performed to investigate the processes of the candidate genes, by applying online tools of the KOBAS 3.0 (https://david.ncifcrf.gov/). The Search Tool for the Retrieval of Interacting Genes (STRING) database (V10.5, https://string-db.org/) was recruited to predict the potential interaction between BNIP3 and apoptosis genes at protein level. The online database of R2: Genomics Analysis and Visualization Platform (https://hgserver1.amc.nl) was applied to determine the clinical survival of the candidate genes. The relative expression of FTO was computed in breast tumor cohort (e.g. IHC samples) compared to the normal cohort, by which the value indicated the number of standard deviations away from the mean of expression in the normal population. High expression: > 1; Low expression: <− 1 (log2).

### Statistical analysis

Means, SD and SEM were analyzed using Graphpad prism 7.0. Two-tailed Student’s t-test, were used to compare the statistical difference between indicated groups. Pearson analysis was used to analyze correlation between genes. Statistical significance was accepted for *P*-values of < 0.05.

## Results

### FTO, an N6-methyladenosine RNA demethylase is up-regulated in human breast cancer

**Fig. 1:**
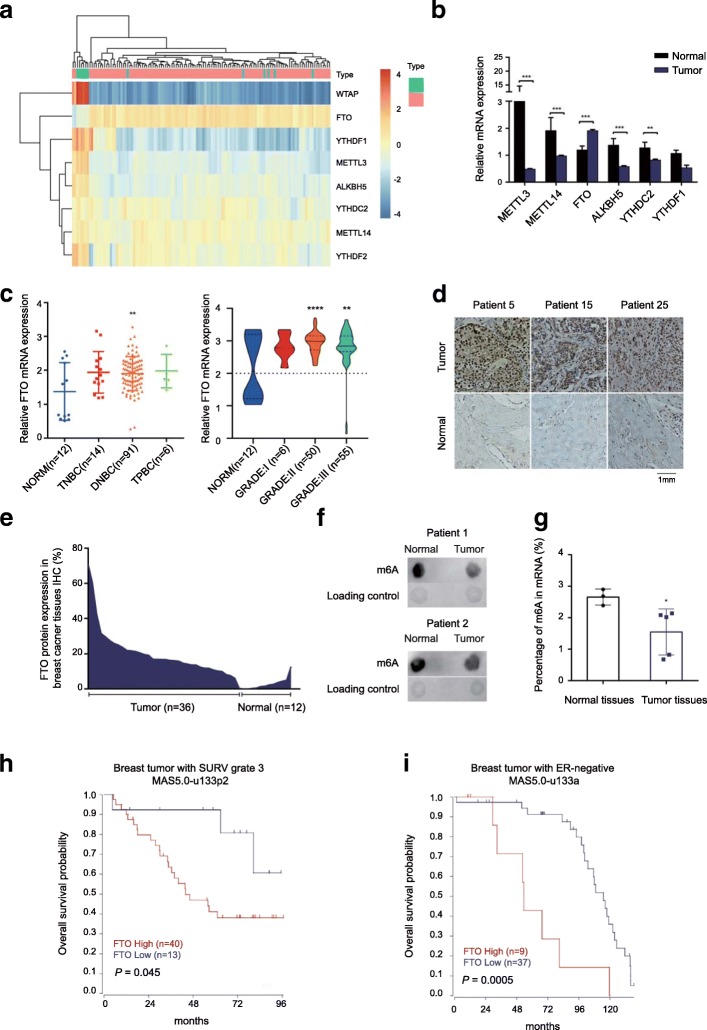
Up-regulation of FTO RNA demethylase in human breast cancer. **a** Heat map diagram of differential gene expression in breast tumors and normal tissues. **b** Expression of the m6A regulatory enzymes in primary human breast tumors. **P ≤ 0.01, ***P ≤ 0.001. **c** Relative FTO mRNA expression level in molecular subtypes and clinical stages of breast tumors. NORM: normal tissues; TNBC: ER−/PR−/Her2-; DNBC: ER−/PR−/Her2+; TPBC: ER+/PR+/Her2+. **P ≤ 0.01, ****P ≤ 0.0001. **d** Higher levels of FTO in human breast cancer tissues in comparison with normal breast tissues by immunohistochemistry assay. **e** FTO up-regulation was quantified from the immunohistochemistry results. **f** The global mRNA m6A level in human breast cancer samples determined by RNA m6A dot-blotting assay. **g** The global mRNA m6A level in human breast cancer samples determined by RNA m6A colorimetric analysis. *P ≤ 0.05. **h** FTO up-regulation was significantly associated with shorter overall survival in patients with advanced stage of breast cancer. **i** FTO up-regulation was significantly associated with shorter overall survival in patients with ER negative breast cancer

To investigate the role of m6A modification in breast cancers, we systematically analyzed the transcriptomic profiles of 111 breast tumors and 12 non-tumorous (NT) breast tissues (GSE9014, Additional file 2: Figure S1A), and identified that FTO, the core m6A demethylase, was significantly up-regulated in breast tumors compared with normal tissue (Fig. 1a and b). We further confirmed the up-regulation of FTO in the group of DNBC (ER−/PR−/Her2+) and late stages (GRADE II and III) three clinical stages of breast cancer (Fig. 1c), suggesting that FTO may play a predominant role in mediating m6A modification in breast cancer. We also found that FTO was higher expressed in breast cancer cell lines than other cancer cell lines (GSE11612, Additional file 2: Figure S1B). To validate the up-regulated RNA level of FTO, we performed the immunohistochemistry (IHC) staining assay to detect the protein expression level of FTO in 36 clinical human breast tumor tissues and 12 corresponding NT adjunct breast tissues (Fig. 1d and Additional file 2: Figure S1C). Consistently, FTO protein was significantly overexpressed in breast tumor tissues compared to their adjunct tissues according to the quantification of IHC results (Fig. 1e), which supported our initial observation of FTO up-regulation in breast cancer. Next, we detected the global m6A level in 2 fresh human breast tumors and their corresponding adjunct NT tissues by the RNA dot-blotting assay (Fig. 1f) and 5 fresh human breast tumors and 3 normal breast tissues by the m6A colorimetric analysis (Fig. 1g). In line with initial observation, a notable decrease of global m6A abundance was detected in breast tumors. Moreover, with clinical outcome analysis, we found that up-regulation of FTO was significantly associated with lower survival rates in patients with advanced stage of breast cancer (Fig. 1h) and patients with ER negative breast cancer (Fig. 1i). It indicates that up-regulation of FTO may be implicated in breast cancer initiation and progression.

### FTO significantly promoted breast cancer cell proliferation, colony formation, and reduced cell apoptosis

**Fig. 2:**
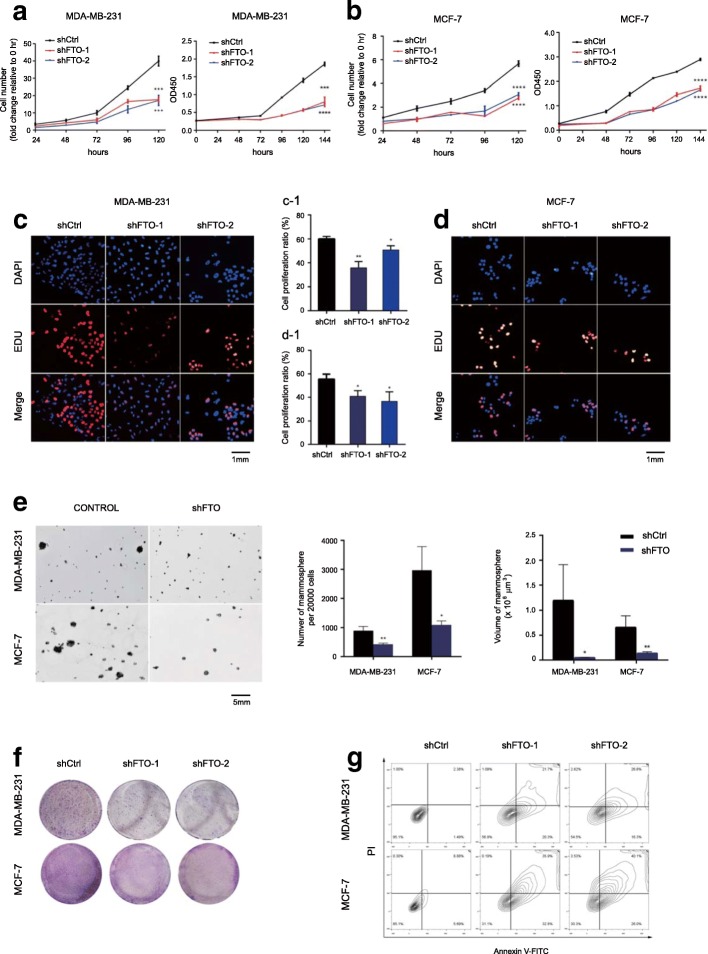
FTO significantly promoted breast cancer cell proliferation, colony formation, and reduced cell death. **a**-**b** Knockdown of FTO significantly reduced cell number and cell growth of MDA-MB-231 cells (**a**) and MCF-7 cells (**b**). ***P ≤ 0.001, ****P ≤ 0.0001. **c**-**d** Cell numbers were counted by FACS (upper panel); Cell growth was determined by CCK8 assay. Mean ± SD. Knockdown of FTO significantly reduced cell proliferation in MDA-MB-231 cells (**c**) and MCF-7 cells (**d**) by Edu cell proliferation assay. **e** Quantification of signal was shown in between as indicated. Knockdown of FTO impaired mammosphere formation of breast cancer cells. Mammospheres were quantified with number and size as indicated. *P ≤ 0.05, **P ≤ 0.01. **f** Knockdown of FTO impaired colony-formation abilities of breast cancer cells. **g** Knockdown of FTO significantly increased cell apoptosis in MDA-MB-231 cells and MCF-7 cells by FACS

To determine whether FTO was critical to breast cancer cell growth, we generated two stable FTO-knockdown models in human MDA-MB-231 and MCF-7 cell lines, by infecting two distinct shRNA lentivirus (shFTO#1 and shFTO#2). The FTO-knockdown effects were confirmed in both RNA expression level and protein expression level (Additional file 3: Figure S2A and B). Knockdown of FTO significant inhibited cell growth in MDA-MB-231 and MCF-7 cells (Fig. 2a and b). We further validated the suppressed effect of FTO knockdown in cell proliferation by the EDU staining assay (Fig. 2c and d). Furthermore, we performed the mammosphere formation assay by seeding control and FTO-knockdown cells in mamosphere culture medium. As shown in Fig. 2e, ~ 1% of total MDA-MB-231 cells and ~ 3% of total MCF-7 cells formed mammospheres containing ~ 900 cells and ~ 2800 cells respectively after 12 days of cultivation in non-adherent dishes. Knockdown of FTO dramatically suppressed mammosphere formation in both MDA-MB-231 cells and MCF-7 cells (Fig. 2e). Similarly, depleting FTO inhibited breast cancer cell colony-forming abilities (Fig. 2f). We next examined the function of FTO in cell survival by flow cytometry with Annexin V/PI staining. FTO depletion resulted in significant cell apoptosis in both MDA-MB-231 and MCF-7 cells (Fig. 2g). Taken together, our results suggest that FTO plays an important role in controlling breast cancer cell growth, colony formation and cell death.

### Silencing FTO inhibited breast tumor growth in vivo

**Fig. 3:**
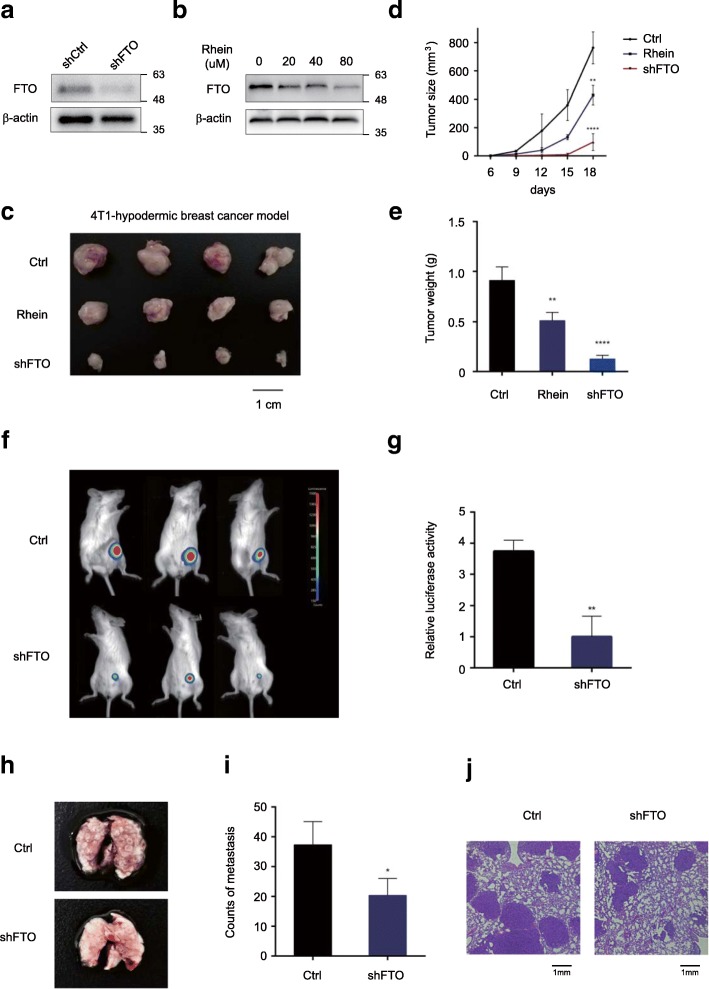
Silencing FTO inhibited breast tumor growth in nude mice models. **a** Stable knockdown of FTO in 4 T1 cells by lentiviral FTO shRNA (shFTO). The knockdown effect was verified at the protein levels. **b** Inhibition of FTO by Rhein in 4 T1 cells with various concentration. **c** Knockdown and inhibition of FTO effectively suppressed 4 T1 cell growth in mice. 1 × 10^6^ 4 T1 cells were subcutaneously implanted in mice. Mice were treated with Rhein or DMSO 3 times per week. **d**, **e** The size (**d**) and the weight (**e**) of tumor formed in the subcutaneous implantation mice model was monitored every 3 days. **P ≤ 0.01, ****P ≤ 0.0001. **f**-**g** Knockdown of FTO dramatically suppressed breast tumor growth in orthotopic xenograft mouse model. Stable FTO-knockdown MDA-MB-231 cells and control cells were injected into the mammary fat pad of each NOD/SCID mouse. 30 days after injection, luciferase activity was measured (**f**) and quantified (**g**) by an IVIS device. **P ≤ 0.01. **h**-**j** Knockdown of FTO abolished lung metastasis in mice. 4 T1 cells were tail vain injected into BALB/c mice. Formation of breast cancer metastatic foci in the lung was pictured (**h**) and quantified (**i**) after 2 weeks. The metastases were confirmed by hematoxylin and eosin staining (**j**). *P ≤ 0.05

To further verify the oncogenic role of FTO in breast cancer, we performed a subcutaneous implantation experiment in BALB/c mice to examine the effect of FTO-knockdown in breast cancer tumorigenicity. Stable FTO-knockdown 4 T1 cells (mouse-derived breast cancer cell line) were constructed by using FTO shRNA (Fig. 3a), and subcutaneously injected into 4-week-old female BALB/c mice. We observed that loss of FTO effectively inhibited breast tumor growth in mice as reflected by the significant reduction of tumor size and tumor weight comparing to the controls (Fig. 3c-e). Alternatively, Rhein, a potent FTO inhibitor [36], was used to examine the inhibitory effect of FTO (Fig. 3b). We intraperitoneally injected Rhein (10 mg/kg) into mice 3 times per week until the tumor reached 10 mm^3^. Consistently, breast tumor retardation was observed in mice treated with Rhein by comparing to the control treated with DMSO (Fig. 3c-e).

In addition, we performed an orthotopic xenograft experiment in NOD/SCID mice. The MDA-MB-231 cells were engineered to stably express luciferase for in vivo imaging. Control and FTO-knockdown MDA-MB-231 cells were orthotopically injected into mouse mammary gland fat pads, and xenograft tumor growth was monitored accordingly. At 30 days after injection, all mice bearing FTO-knockdown cells barely had detected tumors whereas mice bearing control cells showed visible tumor growth as reflected by the luciferase signals in vivo (Fig. 3f and g). To investigate the effect on tumor metastasis, FTO-knockdown 4 T1 cells and control cells intravenously injected into BALB/c mice. As expected, silencing FTO attenuated lung metastasis in mice (Fig. 3h-j). Taken together, our results indicated that FTO plays a critical role in promoting breast tumor growth and metastasis in vivo.

### RNA-Seq analysis identified BNIP3 as a downstream target of FTO-mediated m6A modification

**Fig. 4:**
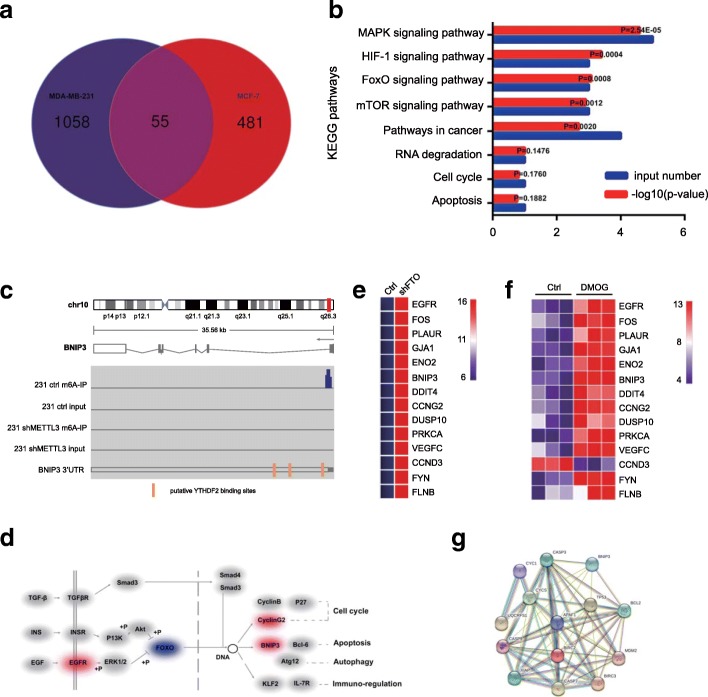
RNA-Seq and m6A-Seq identified BNIP3 as a downstream target of FTO-mediated m6A modification. **a** Venn diagram illustrated overlap in differentially expressed genes in FTO-knockdown MDA-MB-231 cells and MCF-4 cells treated with DMOG. **b** KEGG analysis shows that FTO-knockdown regulate pathways involved in cell proliferation, cell cycle and apoptosis. **c** m6A-Seq identification of m6A modification in BNIP3 mRNA near to the YTHDF2 binding sites. **d** Differentially expressed genes by inhibiting or knockdown of FTO involved in the FoxO signaling pathway. Red color indicates up-regulated genes, while purple color indicates down-regulated genes. **e**, **f** Heatmap of up-regulated genes in FTO-knockdown MDA-MB-231 cells (**e**) and MCF-4 cells treated with DMOG (**f**). **g** Co-expression analysis of BNIP3 by the string

To investigate the molecular mechanism of FTO and identify its downstream targets in breast cancer, we performed transcriptome sequencing to examine the expression changes in our stable FTO-knockdown MDA-MB-231 cells (Additional file 4: Table S2) and MCF-7 cells treated with DMOG, a FTO inhibitor (GSE3188, Additional file 5: Table S3). According to the standard GEO2R analysis and quantile normalization, 55 genes with significant changes were overlapped in these two experiments (Fig. 4a). KOBAS 3.0 was adopted to conduct the gene enrichment pathway in both MDA-MB-231 and MCF-7 cells [37–39]. KEGG analysis revealed that inhibiting FTO could dramatically affect the differentially expressed genes enriched in the signal pathways involved in cell proliferation, cell cycle and apoptosis (Fig. 4b). We also performed the m6A-Seq to map the m6A modification in MDA-MB-231 cells, and found that most m6A signal was enriched around the stop codon of mRNAs. Among these, we selected BNIP3, a pro-apoptosis gene in the FoxO signaling pathway as a candidate target of FTO-mediated m6A modification for further investigation (Fig. 4c and d). BNIP3 was significantly up-regulated in FTO-knockdown MDA-MB-231 cells and FTO-inhibition MCF-7 cells (Fig. 4e and f). The co-expression analysis by the string showed that BNIP3 was closely related with the apoptosis genes such as Bcl-2 and Caspase 3 (Fig. 4g).

### Epigenetic silencing of BNIP3 by an FTO-m6A-dependent mechanism

**Fig. 5:**
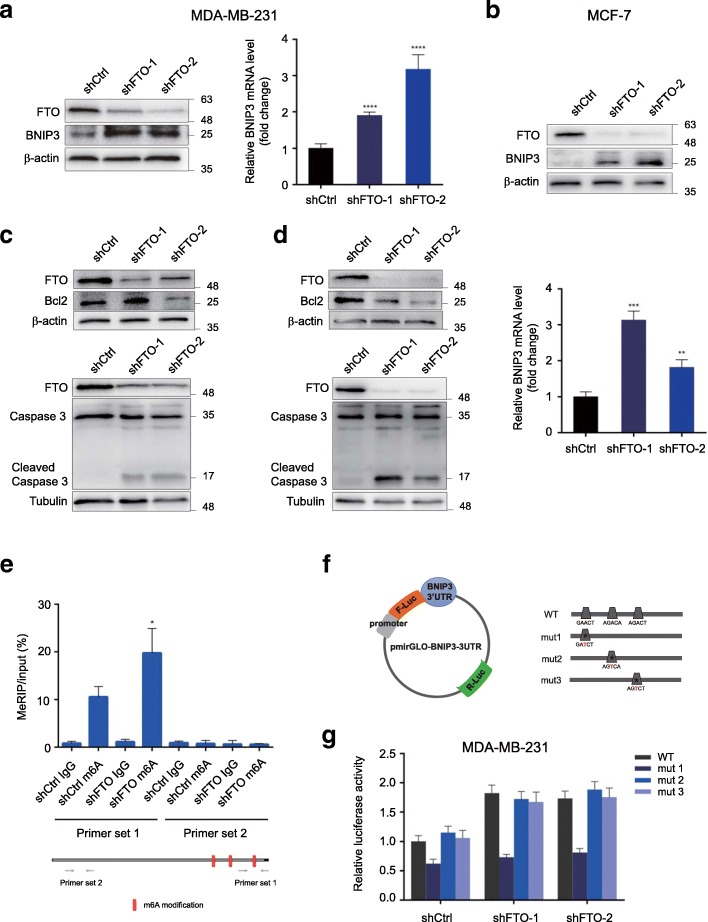
Epigenetic silencing of BNIP3 by an FTO-m6A-dependent mechanism. **a**-**b** BNIP3 expression was significantly up-regulated in both RNA and protein expression level in stable FTO-knockdown MDA-MB-231 cells (**a**) and MCF-7 cells (**b**). **P ≤ 0.01, ***P ≤ 0.001, ****P ≤ 0.0001. **c**, **d** Knockdown of FTO promoted the cleavage of Caaspase 3 and decreased Bcl2 in MDA-MB-231 cells (**c**) and MCF-7 cells (**d**). **e** Knockdown of FTO promoted the m6A methylation in BNIP3 mRNA by the m6A MeRIP analysis. *P ≤ 0.05. **f** Wild-type or m6A consensus sequence mutant BNIP3 3’UTR was fused with firefly luciferase reporter. Mutation of m6A consensus sequences were generated by replacing adenosine with thymine. **g** Relative luciferase activity of the wild-type and 3 mutant BNIP3 3’UTR reporter vectors in FTO-knockdown MDA-MB-231 cells

To verify BNIP3 as a FTO downstream target, we detected both BNIP3 mRNA expression level and protein expression level in breast cancer cells. In agreement with our RNA-seq data, BNIP3 was remarkably up-regulated in stable FTO-knockdown MDA-MB-231 and MCF-7 cells (Fig. 5a and b). Meanwhile, silencing FTO promoted the cleaving of Caspase 3 and decreased the expression of Bcl2 in MDA-MB-231 and MCF-7 cells (Fig. 5c and d). These results showed that FTO inhibited cell apoptosis via down-regulating BNIP3. To validate BNIP3 as a bona fide target of FTO for the m6A modification, we performed the m6A-RNA immunoprecipitation assay and analyzed with qRT-PCR. As expected, knockdown of FTO dramatically promoted the m6A level of BNIP3 mRNA (Fig. 5e). From the m6A RNA-seq, 3 potential m6A sites (RRACH) were identified in the 3′ UTR near to the termination codon of BNIP3 (Fig. 4c). To further prove the effect of m6A modification on BNIP3 expression, we cloned the BNIP3 3’UTR portion containing 3 potential m6A sites into a dual luciferase reporter construct pmirGLO and generated 3 mutant BNIP3 3’UTR reporter vectors, respectively. For the mutant form of BNIP3, we replaced the adenosine base in m6A consensus sequences with thymine to abolish the m6A modification (Fig. 5f). Relative normalized luciferase activities of the wild-type and 3 mutant BNIP3 3’UTR reporter vectors were compared in control and FTO-knockdown MDA-MB-231 cells. A significant induction (~ 1.8 fold) in the luciferase activity was observed in the wild-type 3’UTR of BNIP3 in FTO-knockdown cells compared with control cells, while only mutant #1 almost abolished this induction, indicating the modulation of BNIP3 expression was under the control of FTO-associated m6A modification on site #1 (Fig. 5g). Furthermore, we next investigated the potential mechanism by which m6A methylation regulates the expression of BNIP3. As YTHDF2 is recognized as the main m6A reader in the 3’UTR sites of target genes [40, 41], we measured its role in mediating BNIP3 expression level by m6A. Overexpression of YTHDF2 showed no effects in reducing the mRNA expression level of BNIP3 in FTO-silenced breast cancer cells (Additional file 6: Figure S3A-B), suggesting that FTO mediated-m6A modification decreased BNIP3 expression in the YTHDF2-independent manner.

### BNIP3 is a tumor suppressor and is negatively correlated with FTO expression in clinical breast cancer patients

**Fig. 6:**
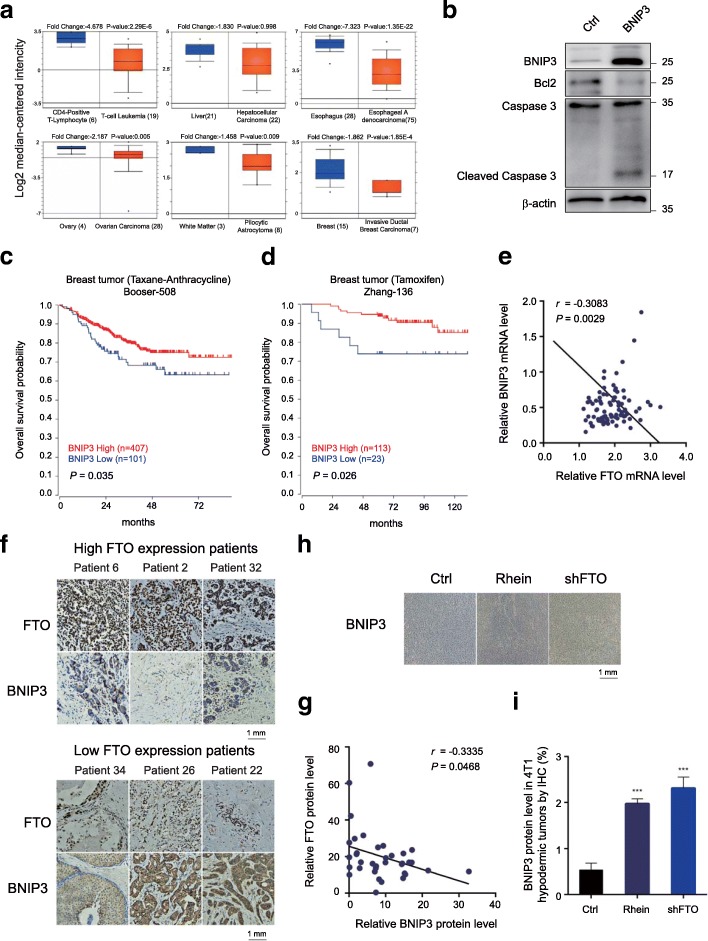
BNIP3 is a tumor suppressor and is negatively correlated with FTO expression in breast cancer. **a** BNIP3 was significantly down-regulated various human cancer including breast cancer tumors comparing to the adjunct tissue. *P ≤ 0.05. **b** Overexpression of BNIP3 activated the cleavage of Caspase 3 and inhibited Bcl2 expression in MCF-7 cells. **c**-**d** Lower expression of BNIP3 was significantly associated with shorter overall survival in patients with breast cancer treated with taxane-anthracycline (**c**) and neuroblastoma (**d**). **e** Correlation analysis of the RNA levels of BNIP3 and FTO in breast tumor samples. **f** Protein expression levels of BNIP3 and FTO by immunohistochemistry assay in human FFPE breast cancer samples. **g** Correlation analysis of the protein levels of BNIP3 and FTO in breast tumor samples based on immunohistochemistry analysis. **h**-**i** BNIP3 expression level in 4T1-hypodermic breast tumor from mice treated with Rhein or FTO shRNA (**h**). The expression level was quantified as indicated (**i**). ***P ≤ 0.001

As a functional target of FTO, BNIP3 is required for cell apoptosis, suggesting its role as tumor suppressor in breast cancer. By analysis of the Oncomine database, we found that BNIP3 was frequently down-regulated in various human cancers including breast cancer comparing to the non-tumor adjunct tissues (Fig. 6a). Overexpression of BNIP3 in MCF-7 cells activated Caspase3 cleavage and inhibited the expression of Bcl-2 (Fig. 6b). Our analysis of R2 datasets revealed that BNIP3 levels are positively correlated with overall survival of patients with breast cancer treated with taxane-anthracycline (*P*-value = 0.035), and with Tamoxifen (*P*-value = 0.026) (Fig. 6c and d). By analyzing the data set (GSE9014), we detected the levels of BNIP3 and FTO in normal and breast tumor samples. A significant negative correlation between BNIP3 and FTO levels was observed in breast tumors (coefficient = − 0.3083, *P* = 0.0029) (Fig. 6e). To validate their correlation, we performed the immunohistochemistry assay to detect the protein levels of BNIP3 and FTO in our breast tumor sample cohort consisting of 36 primary breast tumor tissues. Our results showed that samples with higher FTO expression (vs average expression level of FTO in breast tumor tissues) was frequently associated with lower BNIP3 level and vice versa (Fig. 6f and Additional file 7: Figure S4). Consistently, a significant negative correlation between BNIP3 and FTO levels was observed in our clinical cohort (coefficient = − 0.3325, *P* = 0.0468) (Fig. 6g). Moreover, we also detected that BNIP3 was upregulated in FTO inhibiting or FTO silencing tumor samples in 4T1-hypodermic breast cancer models (Fig. 6h, i). Taken together, we concluded that BNIP3 was negatively correlated with FTO expression in clinical samples.

### Silencing BNIP3 significantly alleviated FTO-dependent tumor growth and metastasis inhibition in vitro and in vivo

**Fig. 7:**
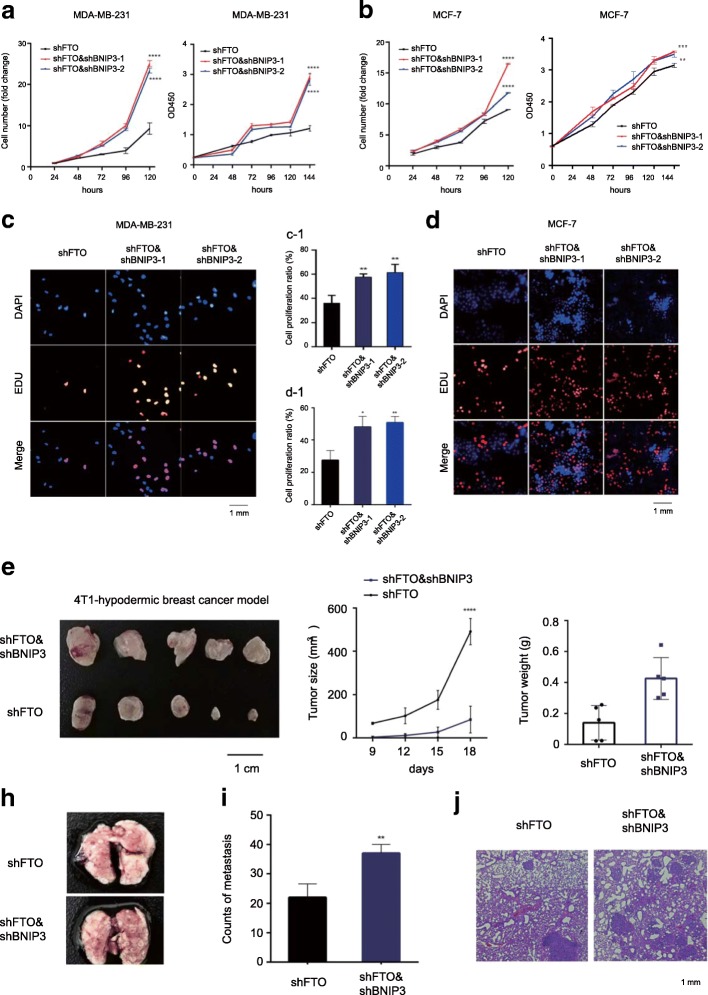
Silencing BNIP3 significantly alleviated FTO-dependent tumor growth and metastasis in vitro and in vivo. **a**, **b** Knockdown of BNIP3 effectively increased cell number and cell growth of FTO-knockdown MDA-MB-231 cells (**a**) and MCF-7 cells (**b**). Cell numbers were counted by FACS (left panel); Cell growth was determined by CCK8 assay (right panel). Mean ± SD. **P ≤ 0.01, ***P ≤ 0.001, ****P ≤ 0.0001. **c**-**d** Knockdown of BNIP3 effectively induced cell proliferation in FTO-knockdown MDA-MB-231 cells (**c**) and MCF-7 cells (**d**) by Edu cell proliferation assay. Quantification of signal was shown in between as indicated. *P ≤ 0.05, **P ≤ 0.01. **e** Knockdown of BNIP3 effectively promoted FTO-knockdown 4 T1 cell growth in mice. FTO-knockdown and double knockdown 4 T1 cells were subcutaneously implanted in nude mice. **f**, **g** The size (**f**) and the weight (**g**) of tumor formed in the subcutaneous implantation mice model was monitored every 3 days. ****P ≤ 0.0001. **h**-**j** Knockdown of BNIP3 promoted lung metastasis in FTO-knockdown mouse model. FTO-knockdown and double knockdown 4 T1 cells were tail vain injected into BALB/c mice. Formation of breast cancer metastatic foci in the lung was pictured (**h**) and quantified (**i**) after 2 weeks. The metastases were confirmed by hematoxylin and eosin staining (**j**). **P ≤ 0.01

To investigate whether BNIP3 mediated FTO-dependent tumor growth and progression, we generated two distinct shRNAs targeting BNIP3 (shBNIP3–1 and shBNIP3–2) in FTO stable knockdown breast cancer cells (Additional file 8: Figure S5A-C). We found that suppression of BNIP3 alleviated the inhibitory effects on cell proliferation mediated by FTO (Fig. 7a and b). Consistently, we confirmed the similar effects of BNIP3 by the EDU staining assay in MDA-MB-231 and MCF-7 cells (Fig. 7c and d). To verify the role of BNIP3 in vivo, we performed subcutaneous implantation with stable FTO-knockdown 4 T1 cells and double knockdown cells (shFTO and shBNIP3) into the 4-week-old female Balb/c mice. Double knockdown of BNIP3 and FTO promoted breast tumor growth in mice as reflected by the significant increase of tumor size and tumor weight comparing to the stable FTO-knockdown cells (Fig. 7e-g). In addition, we detected the increase of lung metastasis in the double knockdown 4 T1 cells by tail vein injection compared to the stable FTO-knockdown 4 T1 cells (Fig. 7h-j). The above results indicated that silencing BNIP3 could significantly alleviate the FTO-dependent inhibitory effects on tumor growth and metastasis in vitro and in vivo.

## Discussion

**Fig. 8:**
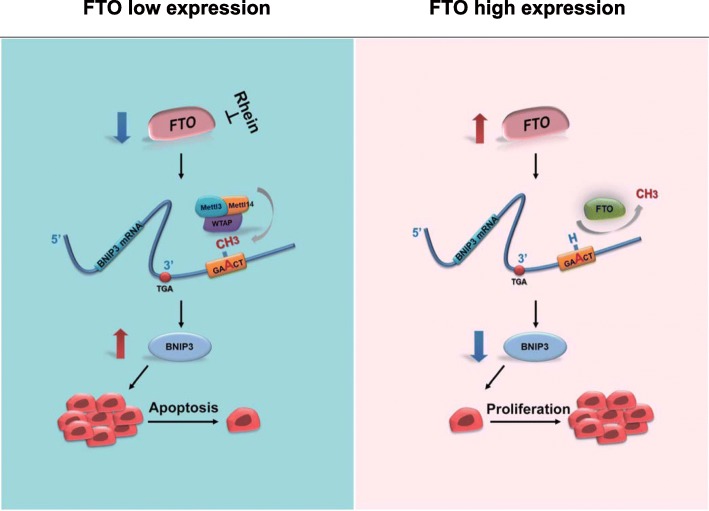
Working model. The oncogenic role of FTO as an m6A demethylase in breast cancer. In FTO low expressed cells, BNIP3 mRNA is m6A methylated at the 3’UTR and up-regulation of BNIP3 induces apoptosis. While in FTO high expression cells, FTO mediates m6A demethylation of BNIP3 to cause its down-regulation, thus promoting cell proliferation. FTO-m6A-BNIP3 signal pathway is considered as a potential therapeutic target for breast cancer therapy

N6-methyladenosine (m6A) modification is the most pervasive modification in human mRNA. Bulks of studies have proved that deregulation of m6A modification was closely related with various human diseases including cancers. FTO, the first and important demethylase of m6A, has been reported as an oncogene in different cancers such as cervical squamous cell carcinoma [17], endometrial cancer [42], etc. However, the roles of FTO in breast cancer initiation and progression are yet fully understood. Here, we show that FTO acted as an oncogene in breast cancer by bursting cell growth and metastasis both in vitro and in vivo. Treatment with Rhein, a FTO inhibitor, decreased tumorigenesis in mice bearing breast tumors. Mechanistically, FTO reduced apoptosis of breast cancer cells at least partially via downregulating expression of BNIP3, a pro-apoptosis gene (Fig. 8). Our findings have revealed a role of FTO in regulation of apoptosis and growth of breast cancer, and theoretically, it suggests that FTO may be a potential therapeutic target for breast cancer.

While we shed light on the epigenetic regulation by FTO in breast cancer, ALKBH5, another m6A demethylase, was previously reported to affect breast cancer stemness phenotype in hypoxia [43]. In their study, they showed that ALKBH5 increased NANOG expression level by demethylating NANOG mRNA in the 3’UTR, promoting breast cancer stem cell renewal. In our study, we systematically analyzed the key regulators of m6A modification in breast cancer, and found that over 70% breast cancer tissues were highly expressed FTO. The global m6A methylation level was decreased in breast tumor tissues by compared to the normal ones. We further showed that high expression level of FTO was significantly related to poor clinic prognosis, indicating the role of FTO in regulation of breast cancer development. Through serial biological functional assays, we have demonstrated that FTO played a pivotal role in promoting cell proliferation, colony formation and metastasis in vitro and in vivo.

Rhein is the first potent FTO inhibitor, which could reversibly bind to FTO catalytic domain and competitively prevent the recognition of m6A modification substrates [44]. In the study of Yan et al., Rhein was proved to increase m6A methylation in leukemia [45]. They showed that combination therapy with Rhein and nilotinibR caused more pronounced recession of leukemia than a single agent treatment in mice. In our present study, we used Rhein to treat the 4T1-hypodermic breast tumor in mice, and found that Rhein could decrease tumor growth to a certain extent although the inhibitory effect was weaker than the tumors treated with shRNA. Development of specific potent FTO inhibitor is considered to be a preferable therapeutic strategy for breast cancers [33].

To decipher the molecular mechanism by which FTO promoted tumor growth in breast cancer, we analyzed the transcriptomic profiling of stable FTO knockdown MDA-MB-231 cells and control cells, MCF-7 cells treated with DMOG (a 2OG competitor which could inhibit FTO activity) and the one treated with DMSO, respectively. From the enrichment analysis, we focused on the FoxO signaling pathway, which was previously reported to be highly correlate with human cancers [46]. FoxO proteins function as transcription factors by controling unchecked neoplastic growth and showing the anti-proliferative and pro-apoptotic effects [47]. Previous study found that FoxO induced cell apoptosis by activating pro-apoptotic Bcl-2 [48, 49]. From our m6A RNA-sequencing analysis, we identified BNIP3, a critical member in the FoxO signaling pathway, was a candidate target of FTO-mediated m6A modification. Silencing FTO promoted BNIP3 expression in both mRNA level and protein level. We identified 3 potential m6A sites in the 3’UTR of BNIP3, and verified that the first site was the essential site to modulate the stability of BNIP3 mRNA by m6A immunoprecipitation assay and luciferase reporter assay. Our results were in line with the an earlier report showing that over 80% of FTO potential targets were negatively regulated by FTO in AML cells as FTO decreased the stability of these mRNA transcripts [29] and another report that m6A editing contributed to the enhancement of HIV-1 mRNA stability [50]. Methylation in the vicinity of stop codons hinted us that the m6A reader YTHDF2 might participate in the regulation of m6A-methylated BNIP3 mRNA stability [50]. YTHDF2 selectively bound to the N-terminal domain of m6A-containing mRNA and located the YTHDF2-mRNA complex to the cellular RNA decay sites [51]. We further investigated whether YTHDF2 affected the BNIP3 mRNA expression level by m6A regulation. We found that overexpression of YTHDF2 had no significant effect in BNIP3 mRNA expression level in FTO-silencing cells, suggesting that FTO mediated m6A demethylation in BNIP3 mRNA to promote it stability via an YTHDF2-independent mechanism. In other words, elevated YTHDF2 expression in FTO silencing breast cancer cells could not inverse the increasing of BNIP3 mRNA expression, indicating the presence of alternative mechanism to stabilize m6A-modified BNIP3 mRNA. And it needs to be further investigated.

To determine whether BNIP3 was the main downstream target of FTO to regulate breast cancer initiation and progression, we generated double knockdown (shBNIP3 and shFTO) breast cancer cell lines. Our results showed that double knockout cells significantly alleviated the inhibitory effects on tumor growth mediated by FTO deficit, proving that FTO promoted breast tumor growth primarily via modulating BNIP3 expression by the m6A modification. Nonetheless, we found all the alleviations were limited that could not recur in the control group, which indicating that the FTO-m6A-BNIP3 signaling pathway may partially explain the effects on breast cancer initiation and progression caused by FTO. It would be very interesting to explore other potential molecular mechanisms involved in epigenetic modulation mediated by FTO.

In summary, we provided compelling evidence that FTO, the key m6A demethylase, was up-regulated in human breast cancer. High expression level of FTO was significantly associated with poor clinical outcome in human breast patients. FTO dramatically promoted breast cancer cell proliferation, colony formation and metastasis through epigenetically down-regulating BNIP3. FTO demethylated m6A in the 3’UTR of BNIP3 and caused its degradation. BNIP3 acted as tumor suppressor and alleviated FTO-dependent tumor growth and metastasis. Altogether, our findings suggest that FTO may serve as a novel potential therapeutic target for breast cancer.

## Additional files

Additional file 1: **Table S1.** Primer lists used in this study. (XLSX 10 kb)

Additional file 2: **Figure S1.** Gene expression in breast cancer. a Volcano plot of gene expression changes in the transcriptome profile of breast tumors and normal tissues. b FTO was higher expressed in breast cancer cell lines than other cancer cell lines compared to the average level of various cancer cell lines, *P ≤ 0.05 . c Immunohistochemistry (IHC) of human normal breast (12 samples) and breast tumor (36 samples) tissues with a specific antibody against FTO. (PDF 230 kb)

Additional file 3: **Figure S2.** Stable knockdown of FTO in breast cancer cells by lentiviral shRNA sequences (shFTO#1 and #2). a The knockdown effect was verified at both the mRNA and protein levels in MDA-MB-231 cells. b The knockdown effect was verified at both the mRNA and protein levels in MCF-7 cells. ***P ≤ 0.001, ****P ≤ 0.0001. (PDF 96 kb)

Additional file 4: **Table S2.** Differentially expressed genes in FTO-knockdown MDA-MB-231 cells. (XLSX 764 kb)

Additional file 5: **Table S3.** Differentially expressed genes in MCF-7 cells treated with DMOG comparing to the ones treated with DMSO. (XLSX 65 kb)

Additional file 6: **Figure S3.** FTO-mediated m6A modification promoted stability of BNIP3 mRNA in an YTHDF2-independent manner. a Overexpression of GFP-Flag-YTHDF2 in MDA-MB-231 cells were analyzed by RT-PCR, ****P ≤ 0.0001. b Measurement of BNIP3 mRNA expression level by overexpression of YTHDF2 in FTO-deficient breast cancer cells. ****P ≤ 0.0001. (PDF 61 kb)

Additional file 7: **Figure S4.** Immunohistochemistry (IHC) of 36 primary human breast tumors FFPE with specific antibodies against BNIP3. (PDF 155 kb)

Additional file 8: **Figure S5.** Stable BNIP3-knockdown in FTO-knockdown MDA-MB-231 cells (a), MCF-7 cells (b) and 4 T1 cells (c) were generated by lentiviral-based shRNA expression. BNIP3 knockdown efficiency was confirmed at the protein levels. (PDF 130 kb)

## Abbreviations

ALKBH5: AlkB homolog 5
AML: Acute myeloid leukemia
ASB2: Ankyrin repeat and SOCS box protein 2
Bcl-2: B-cell lymphoma 2
BNIP3: BCL2 Interacting Protein 3
FTO: Fat mass and obesity-associated
HNRNPA2B1: Heterogeneous Nuclear Ribonucleoprotein A2/B1
IGF2BP: Insulin-like growth factor 2 mRNA-binding protein 2
m6A: N6-methyladenosine
METTL14: Methyltransferase-like 14
METTL3: Methyltransferase-like 3
RARA: Retinoic Acid Receptor Alpha
UTR: Untranslated region
WTAP: Wilms tumor 1 associated protein
