# Supplementary figures and images for: RNA N6-methyladenosine demethylase FTO promotes breast tumor progression through inhibiting BNIP3
Source: Mol Cancer. 2019 Mar 28;18:46. doi: 10.1186/s12943-019-1004-4 (PMC6437932; doi:10.1186/s12943-019-1004-4)

# Supplemental Figure 1 (related to Figure 1)

a

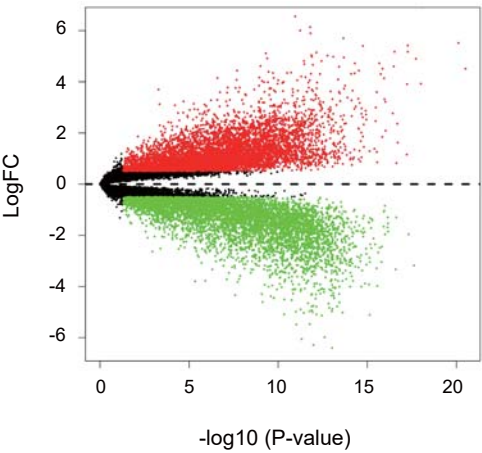

b

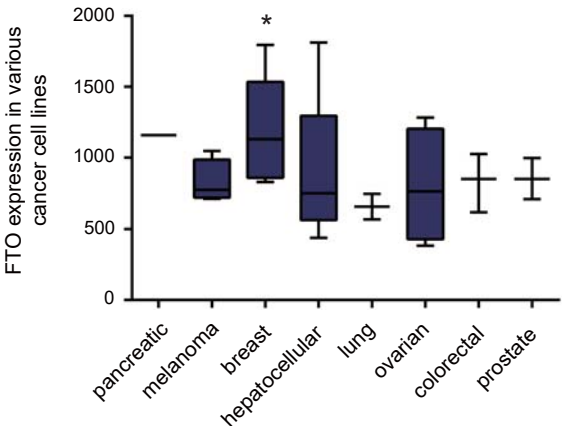

c

36 breast tumor tissues

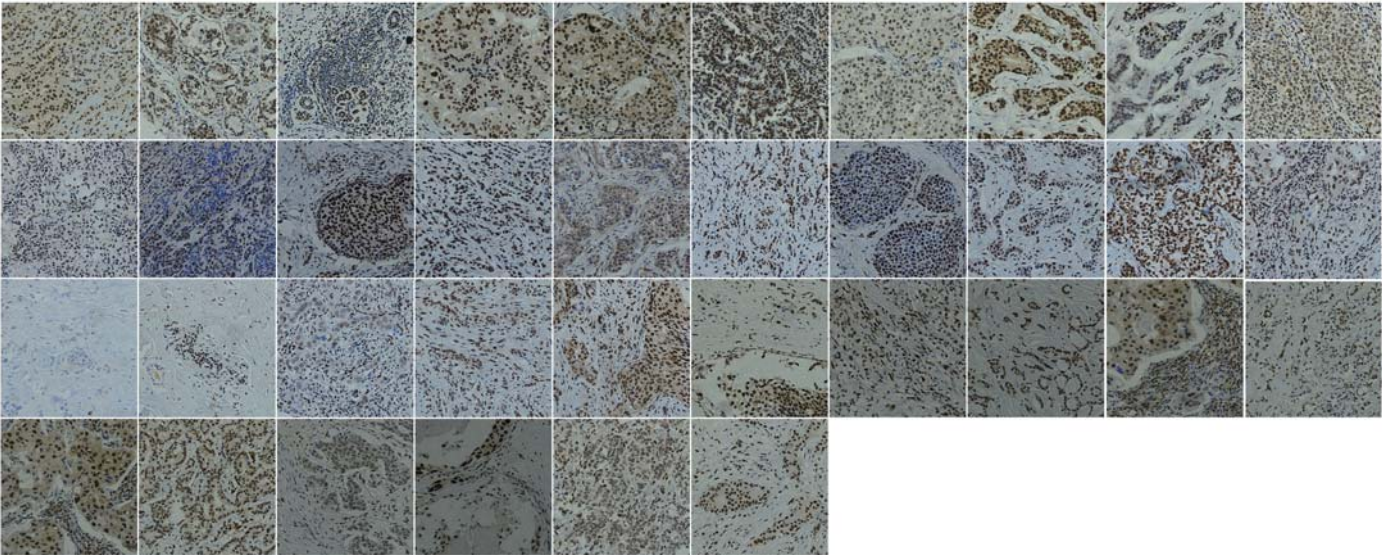

12 breast normal tissues

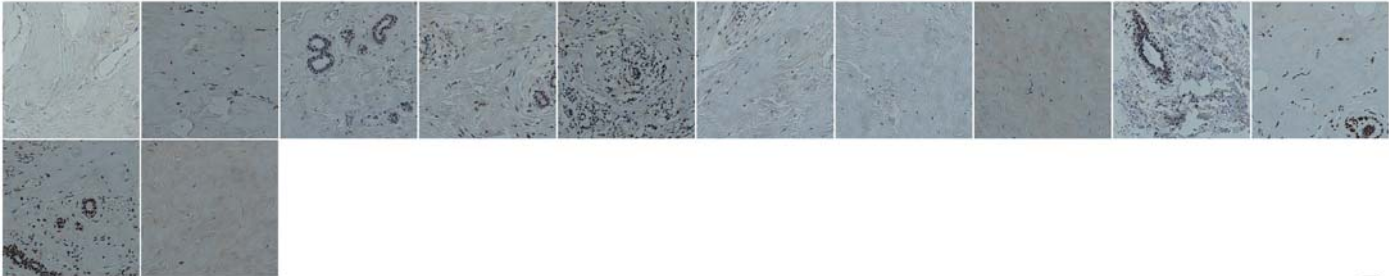

Supplement: Supplementary file 2 — Figure S1. Gene expression in breast cancer. a Volcano plot of gene expression changes in the transcriptome profile of breast tumors and normal tissues. b FTO was higher expressed in breast cancer cell lines than other cancer cell lines compared to the average level of various cancer cell lines, *P ≤ 0.05 . c Immunohistochemistry (IHC) of human normal breast (12 samples) and breast tumor (36 samples) tissues with a specific antibody against FTO. (PDF 230 kb) [file 12943_2019_1004_MOESM2_ESM.pdf]

# Supplemental Figure 2 (related to Figure 2)

a

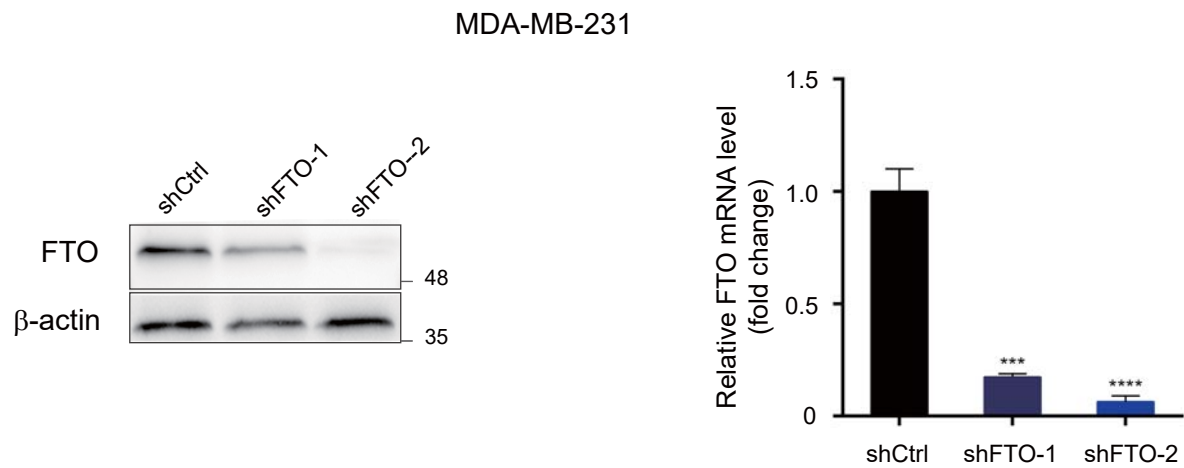

b

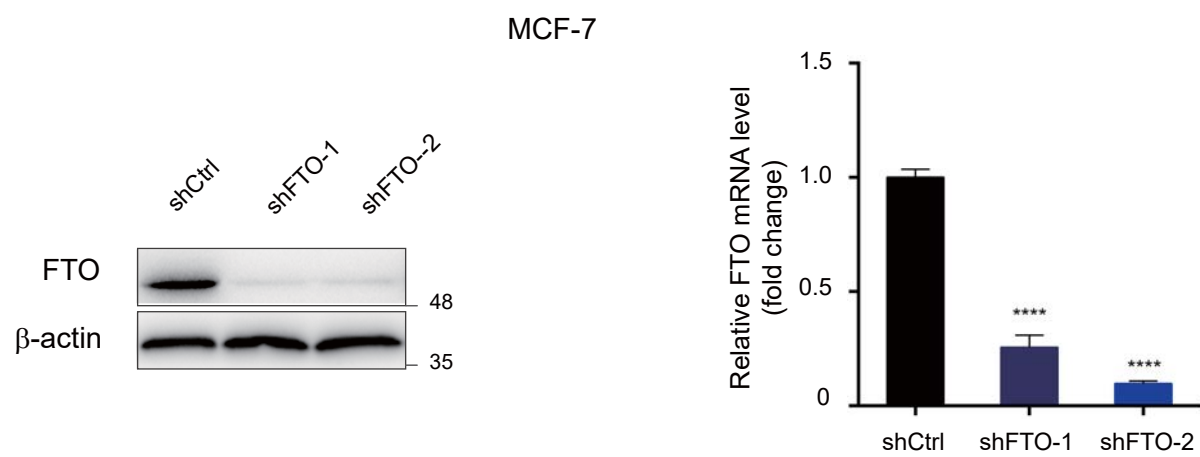

Supplement: Supplementary file 3 — Figure S2. Stable knockdown of FTO in breast cancer cells by lentiviral shRNA sequences (shFTO#1 and #2). a The knockdown effect was verified at both the mRNA and protein levels in MDA-MB-231 cells. b The knockdown effect was verified at both the mRNA and protein levels in MCF-7 cells. ***P ≤ 0.001, ****P ≤ 0.0001. (PDF 96 kb) [file 12943_2019_1004_MOESM3_ESM.pdf]

# Supplemental Figure 3 (related to Figure 5)

a

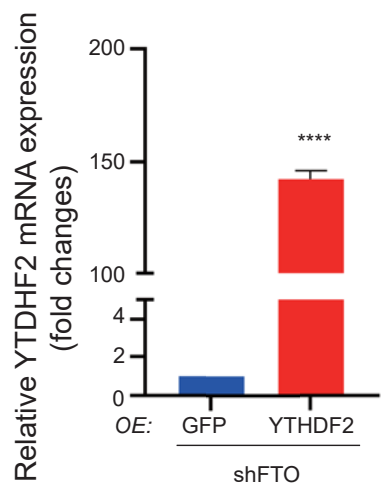

b

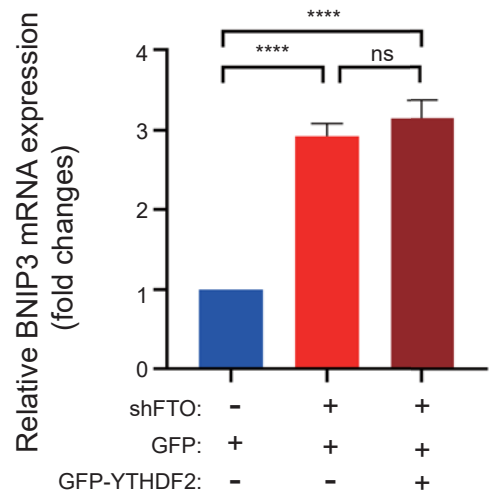

Supplement: Supplementary file 6 — Figure S3. FTO-mediated m6A modification promoted stability of BNIP3 mRNA in an YTHDF2-independent manner. a Overexpression of GFP-Flag-YTHDF2 in MDA-MB-231 cells were analyzed by RT-PCR, ****P ≤ 0.0001. b Measurement of BNIP3 mRNA expression level by overexpression of YTHDF2 in FTO-deficient breast cancer cells. ****P ≤ 0.0001. (PDF 61 kb) [file 12943_2019_1004_MOESM6_ESM.pdf]

# Supplemental Figure 4 (related to Figure 6)

36 breast tumor tissues

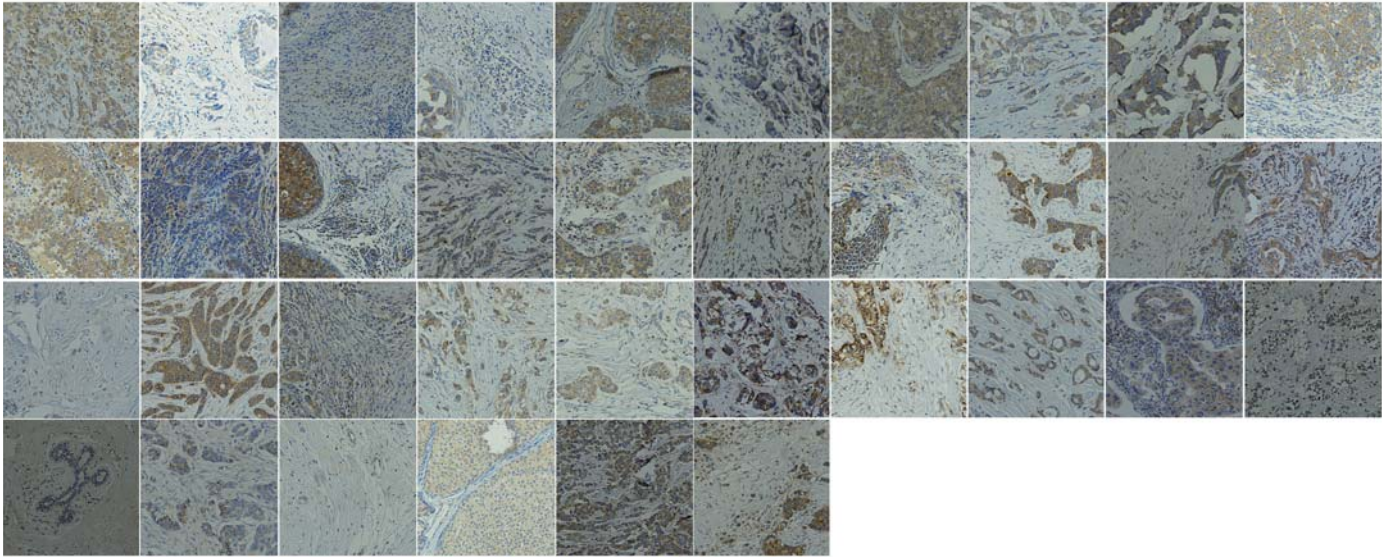

—  
1mm

Supplement: Supplementary file 7 — Figure S4. Immunohistochemistry (IHC) of 36 primary human breast tumors FFPE with specific antibodies against BNIP3. (PDF 155 kb) [file 12943_2019_1004_MOESM7_ESM.pdf]

# Supplemental Figure 5 (related to Figure 7)

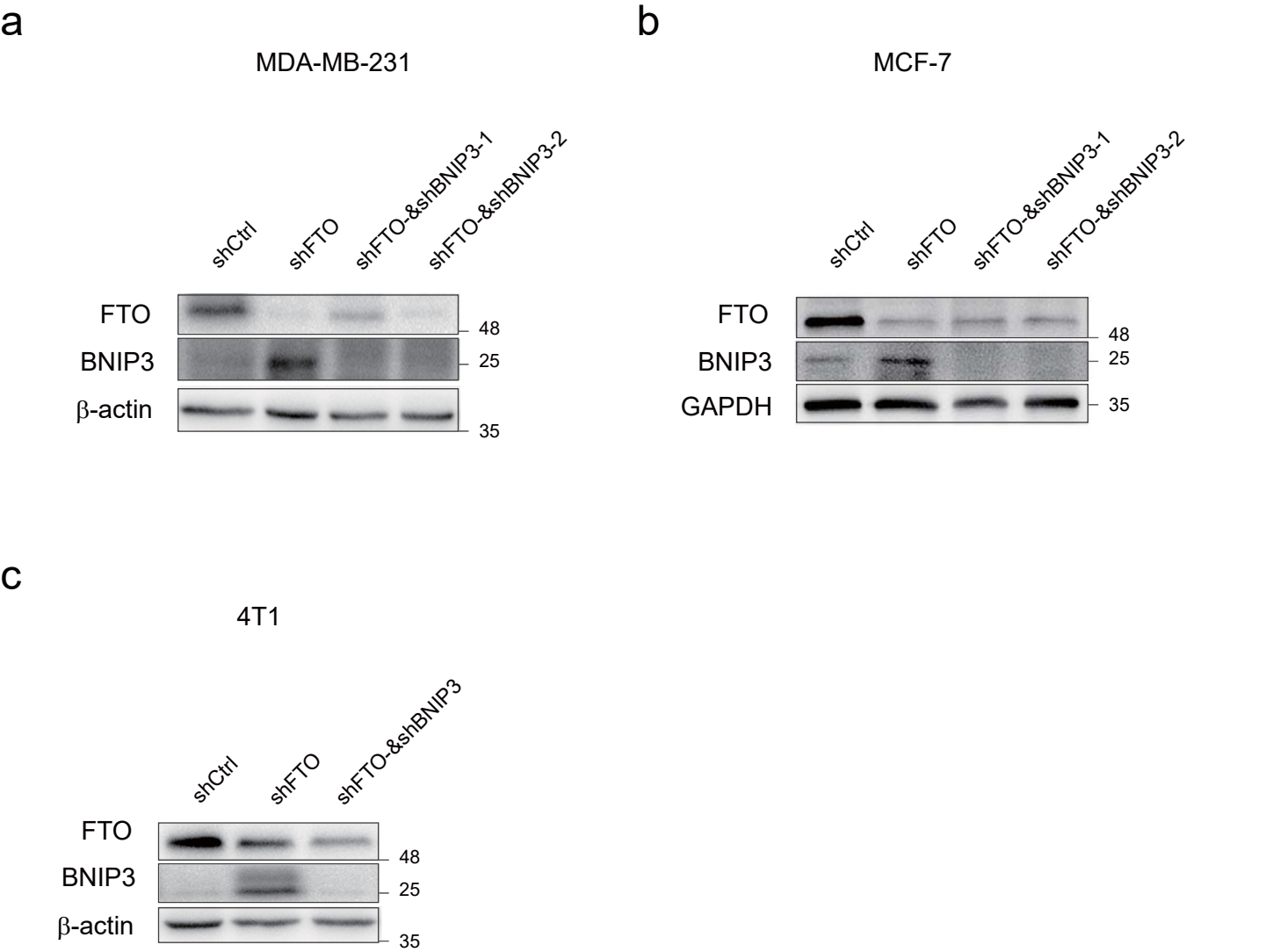

Supplement: Supplementary file 8 — Figure S5. Stable BNIP3-knockdown in FTO-knockdown MDA-MB-231 cells (a), MCF-7 cells (b) and 4 T1 cells (c) were generated by lentiviral-based shRNA expression. BNIP3 knockdown efficiency was confirmed at the protein levels. (PDF 130 kb) [file 12943_2019_1004_MOESM8_ESM.pdf]
